# Exploratory evaluation of encapsulated kratom using multimodal quantitative sensory testing in dogs

**DOI:** 10.3389/fphar.2026.1895945

**Published:** 2026-09-11

**Authors:** Elizabeth A. Maxwell, April Hardison, Raiane A. Moura, Manraj K. Grewal, Erin L. Miscioscia, Veronica Perez-Rodriguez, Fiona Hecker, Diego A. Portela, Judith Bertran, Abhishek Gour, Christopher R. McCurdy, Abhisheak Sharma

**Affiliations:** 1 Pain Innovations Research Lab, Department of Small Animal Clinical Sciences, College of Veterinary Medicine, University of Florida, Gainesville, FL, United States; 2 Translational Drug Development Core, Clinical and Translational Science Institute, University of Florida, Gainesville, FL, United States; 3 Pain Innovations Research Lab, Department of Comparative, Diagnostic, and Population Medicine, College of Veterinary Medicine, University of Florida, Gainesville, FL, United States; 4 Department of Pharmaceutics, College of Pharmacy, University of Florida, Gainesville, FL, United States; 5 Department of Medicinal Chemistry, College of Pharmacy, University of Florida, Gainesville, FL, United States

**Keywords:** analgesia, canine (dog), Mitragyna speciosa, mitragynine, nociceptive thresholds, preclinical canine models, quantitative sensory testing (QST)

## Abstract

**Introduction:**

Kratom has experienced rapidly rising popularity in Western countries, particularly the United States where an estimated 10-20 million individuals use it for management of mental health conditions, performance enhancement, substance use substitution, and chronic pain conditions. Despite widespread anecdotal claims of analgesic benefits, robust preclinical data on kratom’s efficacy remain limited, necessitating translational models to evaluate its potential. The objective of this study was to evaluate the analgesic efficacy of an encapsulated kratom extract compared to buprenorphine and placebo in healthy beagle dogs using quantitative sensory testing.

**Methods:**

Eight healthy female spayed beagle dogs were included in this blinded, randomized, three-phase, crossover design following IACUC approval. Each dog received the following three interventions across the study period: encapsulated kratom extract by mouth at a 1 mg/kg target oral mitragynine dose, an oral placebo, or buprenorphine 0.02 mg/kg intramuscular. Each dog underwent baseline quantitative sensory testing and then at 0.5, 2, 4, 6, and 8 h post-dosing. Three doses of kratom and placebo were administered prior to sensory testing. Sedation scores were also obtained at each time point. Quantitative sensory testing outcomes were analyzed separately by modality using trimmed replicate means, with changes from baseline calculated for each dog and time point. Modality-specific minimum detectable change thresholds, derived from baseline within-time point variability, were used to classify meaningful responses. Treatment effects were evaluated using linear mixed-effects models (treatment, time, and sedation score as fixed effects; dog as a random effect), supplemented by summaries of peak change, time to peak, responder proportions, and trapezoidal area under the curve for both threshold changes and meaningful response burden.

**Results:**

Across QST modalities, buprenorphine elicited the largest threshold increases, with group-average peaks exceeding modality-specific *MDC*_95_ thresholds and high individual-dog responder rates (≥87.5%) in electrical, mechanical, and thermal tests; kratom produced modality-dependent effects, strongest in electrical QST (87.5% responders) and modest in thermal and mechanical.

**Discussion:**

These findings underscore the complex pharmacological profile of kratom, suggesting differential modulation of nociceptive pathways that warrants further investigation into its multiple alkaloids, active metabolites, and their specific contributions to the observed analgesic effect.

## 1 Introduction

With an estimated more than 10 million users in the United States, the consumption of kratom (*Mitragyna speciosa*), a tropical tree indigenous to Southeast Asia, has become increasingly prevalent as a self-prescribed intervention for managing diverse physical, psychological, and social needs (Prozialeck et al., 2012; Eastlack et al., 2020; Henningfield et al., 2024). A significant proportion of users consume kratom to self-treat mental health conditions such as anxiety, depression, post-traumatic stress disorder, and insomnia (Grundmann et al., 2020; Henningfield et al., 2017). Kratom is often used at lower dosages (typically 1–5 g) for its stimulant-like effects to improve daily functioning and productivity (Eastlack et al., 2020; Henningfield et al., 2017). Conversely, higher quantities (typically 5–15 g) are frequently sought for their sedative and analgesic properties, often utilized for the management of opioid withdrawal or as a harm-reduction tool and substitute for other substances such as alcohol or illicit drugs (Eastlack et al., 2020; Henningfield et al., 2024; Prevete et al., 2023). This increased prevalence of self-medication underscores an urgent need for robust scientific investigation into kratom’s purported analgesic efficacy, especially given its complex pharmacological profile (Prozialeck et al., 2012; Eastlack et al., 2020). Recent advancements in human clinical pharmacology have begun to establish a robust safety profile; notably, single ascending dose studies in healthy volunteers have demonstrated that oral doses as high as 12 g of encapsulated powdered leaf, containing approximately 120 mg of mitragynine, are well-tolerated (Huestis et al., 2026; Reissig et al., 2026) While these human trials provide critical data on safety, tolerability, and pharmacokinetics, they often focus on safety endpoints rather than the objective quantification of analgesic utility, or safety in the context of chronic use.

To bridge this gap, translational animal models are essential for investigating the pharmacodynamic relationship between kratom alkaloids and sensory threshold modulation. Dogs serve as a particularly valuable model for this purpose, as they possess similar opioid receptor distributions and pain processing pathways to humans. Such assessments are pivotal for understanding the mechanistic actions and dose-response characteristics of novel analgesics, particularly those with complex alkaloid profiles such as kratom (Grundmann et al., 2018). Quantitative sensory testing (QST) offers a non-invasive, objective approach to evaluate somatosensory function by applying controlled stimuli and measuring pain thresholds (Briley et al., 2013). This methodology is particularly advantageous in preclinical models, as it enables precise characterization of antinociceptive effects across various modalities (Grundmann et al., 2023). Specifically, QST in canine models serves as a crucial indicator of analgesic activity and demonstrates translational value through similarities in somatosensory responses between dogs and humans (Lascelles et al., 2022).

The objective of this study was to compare the analgesic efficacy of encapsulated kratom extract *versus* intramuscular buprenorphine and placebo in healthy beagle dogs, using mechanical, thermal, and electrical QST modalities to quantify changes in sensory thresholds. The authors hypothesize that dogs receiving encapsulated kratom extract will demonstrate increased sensory thresholds compared to placebo, with responses comparable to buprenorphine but accompanied by fewer adverse effects. Plasma mitragynine concentrations are expected to correlate positively with elevations in sensory thresholds.

## 2 Methodology

### 2.1 Study design

#### 2.1.1 Subjects

Eight healthy, spayed, female purpose-bred beagle dogs, aged 5–6 years and weighing 8–10.2 kg, were included following Institutional Animal Care and Use Committee approval (IACUC#202300000196). All dogs were housed in IACUC-approved facilities under temperature- and light-controlled conditions and were maintained in compatible pairs within kennels.

#### 2.1.2 Encapsulated kratom preparation

Encapsulated kratom was formulated specifically for this study under conditions verifying purity, potency, and stability. Each capsule contained 10 mg of mitragynine, standardized from a validated kratom extract. The target mitragynine dose was 1 mg/kg, with exact doses ranging from one to 1.1 mg/kg administered orally *via* capsule on an empty stomach. Kratom was administered every 8 h for three doses prior to testing to establish steady-state pharmacokinetics of mitragynine and assess accumulation. Placebo was administered in a similar fashion.

#### 2.1.3 Experimental phases

The study was a blinded randomized, three-phase, crossover design with a minimum 1-week washout period between phases to ensure complete elimination of study compounds. Each dog received the following three interventions across the study period: an oral placebo, intramuscular buprenorphine (0.02 mg/kg), or encapsulated kratom (mitragynine, 1–1.1 mg/kg), depending on the experimental phase. Dogs were randomized into three groups (A, B, or C) to counterbalance treatments (Table 1).

**TABLE 1:** Dogs were randomized into three groups (A, B, or C). Each dog received the following three interventions across the study period: an oral placebo, intramuscular buprenorphine (0.02 mg/kg), or encapsulated kratom (target mitragynine dose of 1 mg/kg), depending on the experimental phase, in a blinded, randomized, three-phase, crossover design with a minimum 1-week washout between phases.

Dogs will be randomized into three treatment sequences (groups A–C)			
Group	Phase 1	Phase 2	Phase 3
A	Kratom	Buprenorphine	Placebo
B	Buprenorphine	Placebo	Kratom
C	Placebo	Kratom	Buprenorphine

#### 2.1.4 Quantitative sensory testing

**FIGURE 1:**
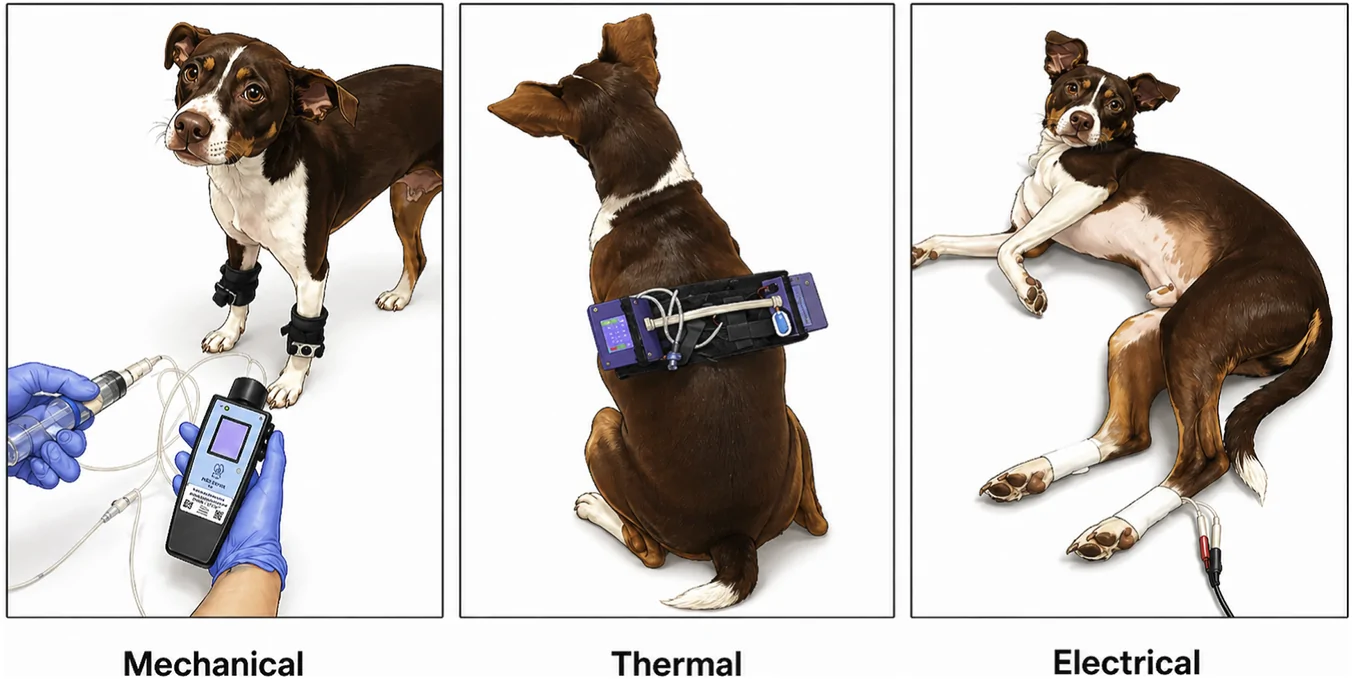
Quantitative sensory testing modalities used to assess sensory thresholds in dogs. Representative illustrations of the three QST modalities evaluated in this study: mechanical pressure algometry, thermal threshold testing, and electrical stimulation. Mechanical testing was performed using a pressure algometer applied to the antebrachium until a conscious behavioral response was observed or the maximum cutoff was reached. Thermal testing used a heat probe device applied to the dog’s dorsal thorax to gradually increase temperature until a response was detected or the thermal cutoff was reached. Electrical testing used surface electrodes over the metatarsals to deliver gradually increasing stimulation until a behavioral response was observed.

Mechanical, thermal (heat), and electrical QST were performed (Figure 1). Prior to study initiation, animals were acclimated to the testing equipment, and training sessions were conducted to ensure consistent identification of true perceptual responses *versus* reflexive movements. All testing was performed in a controlled environment by a trained investigator (EM) to minimize variability and ensure reproducibility. At least 1–2 additional investigators trained in detecting behavioral threshold responses were present. Fur was clipped bilaterally for thermal testing over the dorsal thorax and the metatarsals for electrical testing. Mechanical thresholds were assessed using a pneumatic actuator (Topcat Metrology Ltd., Cambs, UK) that applied incrementally increasing pressure perpendicularly to the skin over the cranial aspect of the antebrachium until an active behavioral response was observed or the maximum cutoff (20 N) was reached. A dummy actuator was placed on the contralateral limb. Thermal thresholds were measured with a heat probe mounted on a jacket (Topcat Metrology Ltd., Cambs, UK) that gradually increased the temperature (2.5 °C/s) until an active response occurred or the 55 °C cutoff was reached. After testing, the area was cooled with an ice pack for 5 min. Aloe was applied to both thermal test sites at the end of the study period. Electrical thresholds were measured *via* a transcutaneous electrical nerve stimulator (TENS) (IntelectVet, Chatanooga, Vista, CA). Pads were placed on either side of the metatarsals and wrapped with an adhesive bandage. A sham wrap was also placed on the contralateral limb. The stimulus intensity was gradually increased until a response was observed, or the maximum of 150 mA was reached. Electrical stimulation parameters consisted of a phase duration of 20 μsec using constant current mode, a cycle time of 10/50, and a frequency of 200 pulses per second (Ruel et al., 2020). For all QST modalities, the threshold was defined as the minimum stimulus intensity eliciting a consistent behavioral withdrawal or nocifensive response. A response or endpoint was defined as deliberate limb withdrawal from the probe, turning toward the stimulated site, vocalization, or application of maximum force. A baseline QST was conducted before the experimental phase to establish individual reference points. Assessments in each phase occurred at 0.5, 1, 2, 4, 6, and 8 h post-third dose in the kratom and placebo groups and post-injection in the buprenorphine group, with at least 60 s between tests to prevent sensitization. For each dog and phase, the initial side was determined randomly by flipping a coin. At each consecutive testing time point, the contralateral side was tested. Testing at each time point was repeated up to three times. If the coefficient of variation exceeded 20%, five replicates were performed, with the lowest and highest values trimmed for analysis. Feasibility was scored for each test from 1 to 5, with 5 indicating high feasibility and 1 indicating low feasibility; if any of the first three scores were 3 or less, five replicates were performed with the highest and lowest values trimmed for analysis. Trials in which the maximum stimulus cutoff was reached without an observable response were scored based on clarity of the non-response.

#### 2.1.5 Sedation scoring

Sedation was assessed at each time point post dose using a standardized composite sedation scoring system adapted from Wagner et al. (Wagner et al., 2017) Parameters evaluated included spontaneous posture, eye position, response to an auditory stimulus, and overall attitude/appearance. Each category was scored on an ordinal scale from 0 (normal/alert) to 3 (marked sedation), yielding a cumulative score ranging from 0 to 12. A consistent observer (EM) performed scoring immediately prior to QST to minimize potential confounding effects of sedation on the interpretation of behavioral responses.

#### 2.1.6 Pharmacokinetic study

Blood samples (1 mL) from the kratom and placebo groups were obtained *via* pre-placed vascular access ports at the following time points: baseline, then trough samples after the first two doses (every 8 h), and then 5 min, 10 min, 20 min, 30 min, then 1, 2, 4, 6, 8, 12, and 24 h, post third dose. In the buprenorphine group, blood samples (1 mL) were obtained pre-dose, then 5 min, 10 min, 20 min, 30 min, then 1, 2, 4, 6, 8, 12, and 24 h. Plasma was harvested and stored at −80 °C until analysis.

Two separate UPLC-MS/MS methods were employed for the quantification of buprenorphine, mitragynine (Avery et al., 2018; Ortiz et al., 2025; Harahap et al., 2022), and their metabolites in canine plasma (Supplementary Table S1). Calibration standards and quality control samples were prepared in pooled canine plasma. The bioanalytical methods achieved a lower limit of quantification (LLOQ) of 0.1 and 1 ng/mL for buprenorphine and mitragynine, respectively. Pharmacokinetic parameters, including maximum plasma concentration (C_max_), time to Cmax (T_max_), area under the plasma concentration-time curve (AUC), half-life (t1/2), volume of distribution/F (Vd/F), and clearance/F (CL/F), were determined using noncompartmental analysis. Pharmacokinetic calculations for the kratom group were performed following the third dose. Samples collected after earlier doses were intended primarily to document accumulation and trough exposure.

Additionally, blood was sampled for a complete blood count (CBC) and biochemical analysis before and after each testing period to evaluate changes related to the safety of drug administration.

#### 2.1.7 Statistical analysis

All analyses were performed in Python using pandas for data handling and stats models for mixed-effects modeling. Outcomes were analyzed separately for each QST testing modality. For each animal and time point, replicate measurements were summarized using a trimmed approach to reduce sensitivity to extreme values: when more than three replicates were available, the minimum and maximum values at that time point were discarded, and the mean of the remaining replicates was used for inferential analyses. Change from baseline was defined as the trimmed mean at each post-dose time point minus the animal’s trimmed mean at baseline for that modality.

Measurement error was characterized by baseline variability. Baseline replicate sets were trimmed as described above, and the within-timepoint standard deviation was computed. For each modality, baseline SDs were pooled across animals and time points to estimate typical within-timepoint variability; this pooled SD was then used to compute the standard error of measurement. The minimum detectable change at 95% confidence was defined as $MDC_{95}=1.96\times \sqrt{2}\times SEM$ . The resulting modality-specific $MDC_{95}$ values were: electrical, 4.58 mA; mechanical, 2.72 N; and thermal, 3.30 °C.

For responder-type summaries, an observation was classified as a “meaningful change” if the change from baseline ( Δ ) was $\geq MDC_{95}$ and the absolute trimmed mean exceeded a modality-specific criterion threshold. Electrical outcomes used the $MDC_{95}$ criterion only as none of the dogs reached maximal threshold cut-off.

The primary analysis employed linear mixed-effects models fit to Δ within each modality. Fixed effects included treatment, time, and sedation score as a covariate. Sedation scores were standardized within each modality, such that the sedation coefficient reflected the expected change in Δ per 1 SD increase in sedation score. Random intercepts for animals accounted for repeated measures within animals.

To provide time-integrated and peak-effect summaries consistent with the repeated-measures outcomes, animal-level trapezoidal area under the curve of Δ was computed over the post-dose period for each modality. Additionally, a “meaningful response burden” was calculated as the trapezoidal AUC of the binary meaningful-change indicator over time (in hours above threshold). Peak Δ and time to peak were summarized descriptively by treatment and modality. Responder effects were summarized as absolute risk differences relative to placebo, representing the percentage-point increase in dogs exceeding the modality-specific $MDC_{95}$ threshold. Duration of meaningful response was summarized as the difference in trapezoidal AUC of the binary responder indicator relative to placebo, expressed as hours above $MDC_{95}$ .

Given that this research represents an exploratory, first-in-species evaluation of encapsulated kratom’s analgesic utility in a canine model, a formal *a priori* sample size calculation was not performed due to the lack of existing data regarding the expected effect size and variance of kratom alkaloids on canine QST modalities. Therefore, a sample size of eight dogs was selected based on feasibility, ethical animal use considerations, and the efficiency of the randomized, blinded, crossover design. Because of the small sample size (n = 8 dogs) and the number of paired comparisons performed across modalities and derived endpoints, paired tests were considered exploratory. Raw p-values are reported for transparency, and multiplicity-adjusted p-values were calculated using Benjamini–Hochberg FDR correction.

## 3 Results

**FIGURE 2:**
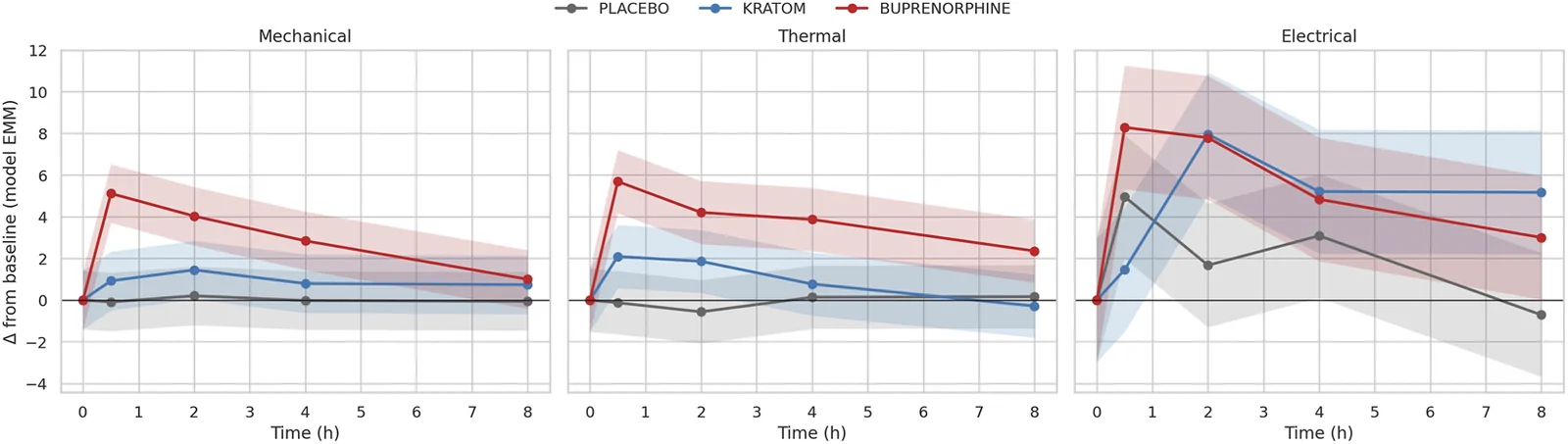
Change from baseline sensory thresholds following placebo, encapsulated kratom, and buprenorphine administration. Mean change from baseline (Δ) sensory thresholds are shown over time for mechanical, thermal, and electrical quantitative sensory testing modalities. Dogs received placebo, oral encapsulated kratom, and intramuscular buprenorphine in a randomized crossover design. Lines represent treatment-specific mean responses, and shaded regions represent standard deviation. Buprenorphine produced the most consistent increases in sensory thresholds across all three modalities, with marked elevations in mechanical, thermal, and electrical thresholds. Kratom produced its most prominent threshold elevation in the electrical modality, with more modest changes observed in mechanical and thermal thresholds.

Across QST modalities, buprenorphine produced the largest overall increase in sensory thresholds, with group-average peak responses exceeding the modality-specific $MDC_{95}$ in all modalities and high rates of individual-dog exceedance. Kratom showed a modality-dependent pattern, with the clearest signal in electrical QST and smaller effects in mechanical and thermal modalities (Figure 2). The per-modality summaries of peak, AUC, % exceeding $MDC_{95}$ by peak, and AUC above $MDC_{95}$ can be found in Table 2. Placebo-anchored responder analysis demonstrated the strongest treatment effect for buprenorphine, particularly for mechanical and thermal QST. Kratom showed a more modest placebo-adjusted responder effect, with the clearest signal observed for mechanical testing. Placebo-anchored responder effects and uncertainty intervals for all modalities are summarized in Table 3. Across all modalities and treatment groups, the median feasibility score was 5.0 with an IQR of 5.0–5.0, indicating that responses were generally easy to interpret. The proportion of scores ≤3 was low across the dataset, with the highest value in the electrical placebo group at 4.4% (Table 4).

**TABLE 2:** Per-modality summary of treatment effects on sensory threshold change from baseline. Peak mean change from baseline, peak standard deviation (SD), trapezoidal area under the curve (AUC) for change from baseline, AUC SD, percentage of dogs exceeding the modality-specific minimum detectable change at 95% confidence ( $MDC_{95}$ ) by peak response, and AUC above $MDC_{95}$ are summarized for each quantitative sensory testing modality and treatment group. $MDC_{95}$ thresholds were calculated separately for each modality based on baseline within-timepoint variability and were used to identify responses exceeding expected measurement error. Higher peak and AUC values indicate greater magnitude and duration of sensory threshold elevation. AUC above $MDC_{95}$ reflects the time-integrated burden of meaningful threshold change over the post-treatment observation period.

Modality	Treatment	Peak mean	Peak sd	Auc mean	Auc sd	% exceed	Auc above mean	Auc above sd
Mechanical	Buprenorphine	7.15	5.23	22.68	13.15	87.50	10.48	13.15
Mechanical	Kratom	2.32	0.83	7.28	5.53	37.50	0.38	0.55
Mechanical	Placebo	0.83	0.68	0.08	6.84	0.00	0.00	0.00
Thermal	Buprenorphine	6.40	2.46	29.36	19.71	87.50	12.40	11.21
Thermal	Kratom	2.86	2.39	7.07	16.22	37.50	2.30	3.93
Thermal	Placebo	1.55	2.07	−0.37	13.36	25.00	0.61	1.59
Electrical	Buprenorphine	10.50	3.33	42.43	18.89	87.50	17.22	11.85
Electrical	Kratom	9.88	6.88	41.34	29.59	87.50	20.85	25.11
Electrical	Placebo	7.17	4.10	15.71	19.37	75.00	5.18	5.40

Units for peak Δ and AUC, of Δ are modality-specific: electrical = mA and mA·h; mechanical = N and N·h; thermal = °C and °C·h. AUC, above MDC95 is reported as hours above threshold.

**TABLE 3:** Placebo-anchored responder and duration effects across quantitative sensory testing modalities. Responder effects are reported as absolute risk difference (RD) *versus* placebo, representing the percentage-point difference in the proportion of dogs exceeding the modality-specific minimum detectable change at 95% confidence ( $MDC_{95}$ ) at any post-treatment time point. Duration effects are reported as the placebo-adjusted difference in mean time above $MDC_{95}$ , expressed as hours above thresholds. Positive values indicate greater responder probability or longer duration of meaningful sensory threshold elevation compared with placebo.

Modality	Treatment	RD any vs. placebo	RD 95CI	Diff mean duration h vs. placebo	Diff mean duration 95CI
Mechanical	Kratom	0.375	4 to 71 pp	0	0.0 to 0.0
Mechanical	Buprenorphine	0.875	65 to 110 pp	0.8125	0.19 to 1.69
Thermal	Kratom	0.125	−33 to 58 pp	0.625	0.0 to 1.5
Thermal	Buprenorphine	0.625	25 to 100 pp	2.9375	1.44 to 4.56
Electrical	Kratom	0.125	−25 to 50 pp	1.5	−0.19 to 3.19
Electrical	Buprenorphine	0.25	−5 to 55 pp	0.625	−0.81 to 2.0

**TABLE 4:** Feasibility of quantitative sensory testing response interpretation in beagles administered placebo, kratom, or buprenorphine. Feasibility was scored using a 5-point ordinal scale, where one indicated that the response was difficult to interpret and five indicated that the response was easy to interpret. Scores from individual assessments were pooled across dogs and post-dosing time points within each modality and treatment group. Values are reported as median feasibility score, interquartile range, and the percentage of scores less than or equal to three, representing responses with lower interpretability.

​	Modality	Treatment group	Median feasibility score	IQR	Scores ≤3, n/N	% feasibility ≤3
0	Mechanical	Placebo	5.0	5.0–5.0	2/166	1.2
1	Mechanical	Kratom	5.0	5.0–5.0	5/179	2.8
2	Mechanical	Buprenorphine	5.0	5.0–5.0	2/176	1.1
3	Thermal	Placebo	5.0	5.0–5.0	2/135	1.5
4	Thermal	Kratom	5.0	5.0–5.0	0/138	0.0
5	Thermal	Buprenorphine	5.0	5.0–5.0	1/140	0.7
6	Electrical	Placebo	5.0	5.0–5.0	7/160	4.4
7	Electrical	Kratom	5.0	5.0–5.0	2/149	1.3
8	Electrical	Buprenorphine	5.0	5.0–5.0	5/142	3.5

### 3.1 Mechanical QST

Mechanical QST showed a strong buprenorphine effect and a smaller kratom effect compared to the buprenorphine group. Mean peak Δ was 7.15 N under buprenorphine and 2.32 N under kratom, compared with 0.83 N under placebo. The proportion of dogs exceeding mechanical $MDC_{95}$ = 2.72 N by peak was 87.5% for buprenorphine, 37.5% for kratom, and 0.0% for placebo. Mean AUC Δ was 22.68 N·h for buprenorphine, 7.28 for kratom, and 0.08 N·h for placebo.

Paired tests vs. placebo indicated significant within-dog increases for buprenorphine in mechanical peak (mean paired difference +6.32, p = 0.0127) and mechanical AUC (mean paired difference +22.60, p = 0.0041). The buprenorphine effect on mechanical AUC above $MDC_{95}$ was positive but did not reach p < 0.05 (mean paired difference +10.48, p = 0.0589). Kratom also showed statistically significant paired increases vs. placebo for mechanical peak (mean paired difference +1.49, p = 0.0056) and mechanical AUC (mean paired difference +7.20, p = 0.0492), which remained FDR-significant, but the magnitude was smaller and the AUC-above- $MDC_{95}$ endpoint remained low and not significant (mean paired difference +0.38, p = 0.0934), consistent with limited $MDC_{95}$ exceedance under kratom in mechanical QST.

### 3.2 Thermal QST

Thermal responses increased most strongly for buprenorphine, with comparatively modest and inconsistent responses observed in kratom dosed animals. Mean peak delta was 6.40 °C with buprenorphine, 2.86 °C under kratom, and 1.55 °C under placebo. The proportion of dogs exceeding thermal $MDC_{95}$ = 3.30 by peak was 87.5% for buprenorphine, 37.5% for kratom, and 25.0% for placebo. Mean AUC Δ was 29.36 °C·h for buprenorphine, 7.07 °C·h for kratom, and −0.37 °C·h for placebo. Mean AUC above $MDC_{95}$ was 12.40 h for buprenorphine, 2.30 h for kratom, and 0.61 h for placebo.

Paired tests vs. placebo showed significant within-dog increases for buprenorphine in thermal peak (mean paired difference +4.85, p = 0.0061), thermal AUC (mean paired difference +29.73, p = 0.0081), and thermal AUC above $MDC_{95}$ (mean paired difference +11.79, p = 0.0245), and remained significant after FDR correction. Kratom did not show statistically significant within-dog differences vs. placebo in thermal peak (mean paired difference +1.31, p = 0.2478), thermal AUC (mean paired difference +7.44, p = 0.2807), or thermal AUC above $MDC_{95}$ (mean paired difference +1.68, p = 0.1713), however it is important to note that the study was underpowered for detecting differences between kratom and placebo in thermal modality.

### 3.3 Electrical QST

Electrical responses were elevated under both active treatments. Mean peak delta was 10.50 mA under buprenorphine, 9.87 mA under kratom, and 7.17 mA under placebo. The proportion of dogs with peak Δ exceeding electrical $MDC_{95}$ = 4.58 mA was 87.5% for buprenorphine, 87.5% for kratom, and 75.0% for placebo. Mean AUC Δ was 42.43 mA·h for buprenorphine, 41.34 mA·h for kratom, and 15.71 mA·h for placebo. Mean AUC above $MDC_{95}$ was 17.22 h for buprenorphine, 20.85 h for kratom, and 5.18 h for placebo.

In paired testing vs. placebo, buprenorphine showed statistically significant within-dog increases in electrical peak (mean paired difference +3.33, p = 0.0202) and electrical AUC (mean paired difference +26.72, p = 0.0229); as well as electrical AUC above $MDC_{95}$ (mean paired difference +12.04, p = 0.0155). The AUC effect remained significant after all-test FDR correction. In contrast, kratom’s electrical increases were directionally positive but not statistically significant in paired testing for electrical peak (mean paired difference +2.71, p = 0.3629), electrical AUC (mean paired difference +25.64, p = 0.1382), and electrical AUC above $MDC_{95}$ (mean paired difference +15.68, p = 0.1105), reflecting larger within-dog variability for this contrast despite similar group means.

### 3.4 Sedation scoring

**FIGURE 3:**
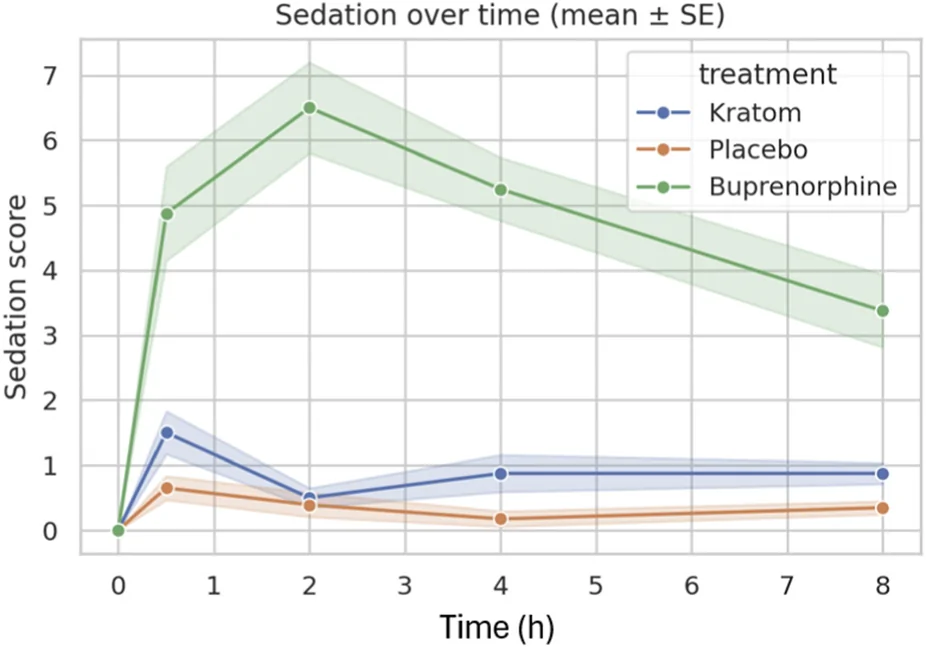
Sedation scores over time following placebo, encapsulated kratom, and buprenorphine administration. Mean sedation scores are shown over time for dogs receiving placebo, oral encapsulated kratom, or intramuscular buprenorphine in a randomized crossover design. Points represent treatment-specific mean sedation scores, and shaded regions represent standard error. Buprenorphine was associated with substantially higher sedation scores across the post-administration period. In contrast, placebo and kratom were associated with minimal sedation, with kratom producing only mild, transient increases in sedation scores.

Sedation scores were near zero for the placebo group, modest for kratom, and markedly elevated for buprenorphine, consistent with a sedation-driven upward shift in thresholds that partially overlaps with the analgesic signal (Figure 3). Sedation scores in this dataset were clustered at 0 for placebo and kratom in many observations but diverged strongly for buprenorphine. For example, in placebo, sedation averaged 0.38 (max 3), kratom averaged 0.75 (max 4), and buprenorphine averaged 4.00 (max 11). To evaluate whether sedation influenced apparent QST treatment effects, treatment–placebo contrasts were re-estimated with sedation score included as a covariate. Buprenorphine-associated threshold increases were attenuated after sedation adjustment, particularly for electrical and mechanical testing. For electrical QST, the buprenorphine–placebo difference at 2 h decreased from 8.29 mA unadjusted to 1.69 mA after sedation adjustment, and at 4 h decreased from 3.92 mA to −3.78 mA. Similarly, the mechanical buprenorphine–placebo difference at 0.5 h decreased from 5.13 N to 1.67 N, and at 2 h decreased from 3.75 N to 0.54 N. In contrast, kratom-associated electrical effects were minimally attenuated after sedation adjustment, with the kratom–placebo difference remaining positive at 2 h (8.33 mA unadjusted and adjusted) and 8 h (7.92 mA unadjusted; 7.41 mA adjusted). Full adjusted and unadjusted treatment estimates are provided in Supplementary Table S2.

### 3.5 Pharmacokinetic analysis and exposure response analysis

**FIGURE 4:**
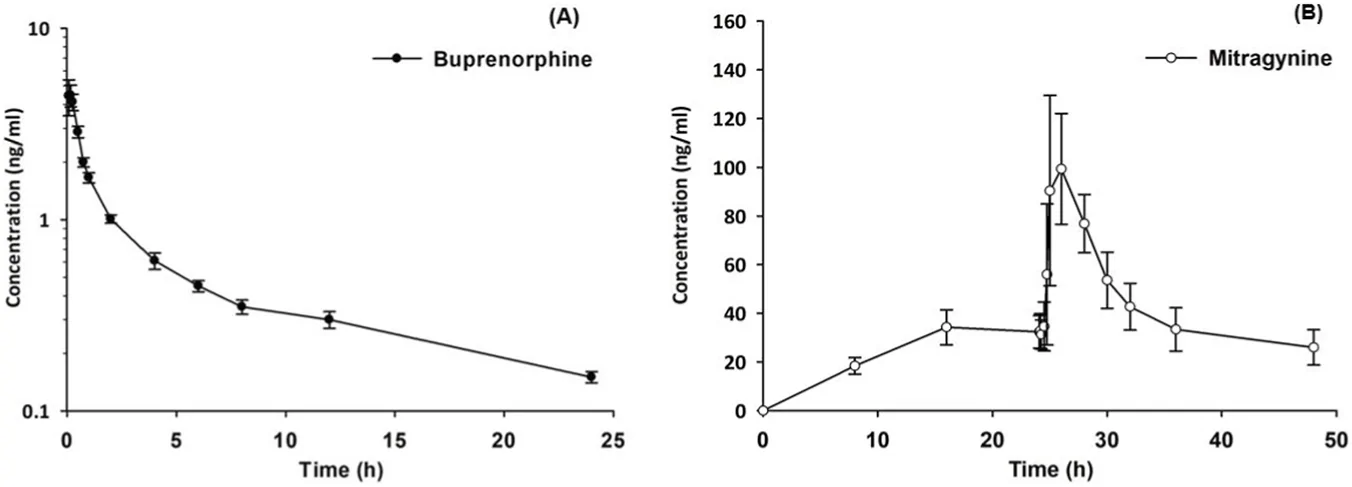
Mean plasma-concentration time profile of buprenorphine after intramuscular administration (0.02 mg/kg) in beagles **(A)** and mitragynine after oral dose (1 mg/kg) in beagles **(B)**. Data are represented as mean ± SEM (n = 8, each time point). Blood samples were obtained at the following time points: baseline, then trough samples after the first two doses (every 8 h), and then 5 min, 10 min, 20 min, 30 min, then 1, 2, 4, 6, 8, 12, and 24 h, post third dose.

Following intramuscular administration of buprenorphine at a dose of 0.02 mg/kg, C_max_ was 4.9 ng/mL. The area under the plasma concentration-time curve up to 24 h (AUClast) was 11.4 ng·h/mL. The terminal elimination half-life (t_1/2_) of buprenorphine was 13.5 h, demonstrating that the compound remained in systemic circulation for an extended duration following administration. The metabolites of buprenorphine (norbuprenorphine, norbuprenorphine-3-glucuronide, and buprenorphine-3-glucuronide) were below LLOQ. Following oral dose of mitragynine (1 mg/kg), dogs reached a C_max_ of 133.3 ng/mL post third dose. The compound showed a T_max_ of 2.4 h and t_1/2_ of 18.7 h. Mean plasma-concentration-time profile and calculated pharmacokinetic parameters are presented in Figure 4 and Table 5. Plasma concentrations of additional kratom alkaloids and metabolites were generally low and insufficient to support reliable noncompartmental pharmacokinetic parameter estimation. Therefore, pharmacokinetic analyses were limited to mitragynine. Measured alkaloids and metabolites are provided in Supplementary Table S1.

**TABLE 5:** Pharmacokinetic parameters of buprenorphine and mitragynine in plasma after intramuscular and oral dose at 0.02 and 1 mg/kg in beagles, respectively.

Parameters	Buprenorphine	Mitragynine^a^
C_max_ (ng/mL)	4.9 ± 0.7	133.3 ± 29.9
T_max_ (h)	0.13 ± 0.02	2.4 ± 0.5
AUC_last_ (ng.h/mL)	11.4 ± 0.6	1,050.9 ± 243.2
Cl/F (L/h/kg)	1.34 ± 0.05	—
V_d_/F (L/kg)	26.5 ± 2.5	—
T_1/2_ (h)	13.8 ± 1.3	18.7 ± 1.1

Data are presented as mean ± SEM (n = 8).

C_max_, maximum plasma concentration; T_max_, time to reach C_max_; AUC_last_, area under the curve for plasma concentration up to 24 h, Cl/F, clearance after intramuscular administration; V_d_/F, volume of distribution after intramuscular administration; T_1/2_, elimination half-life.

^a^ Post-third dose.

**FIGURE 5:**
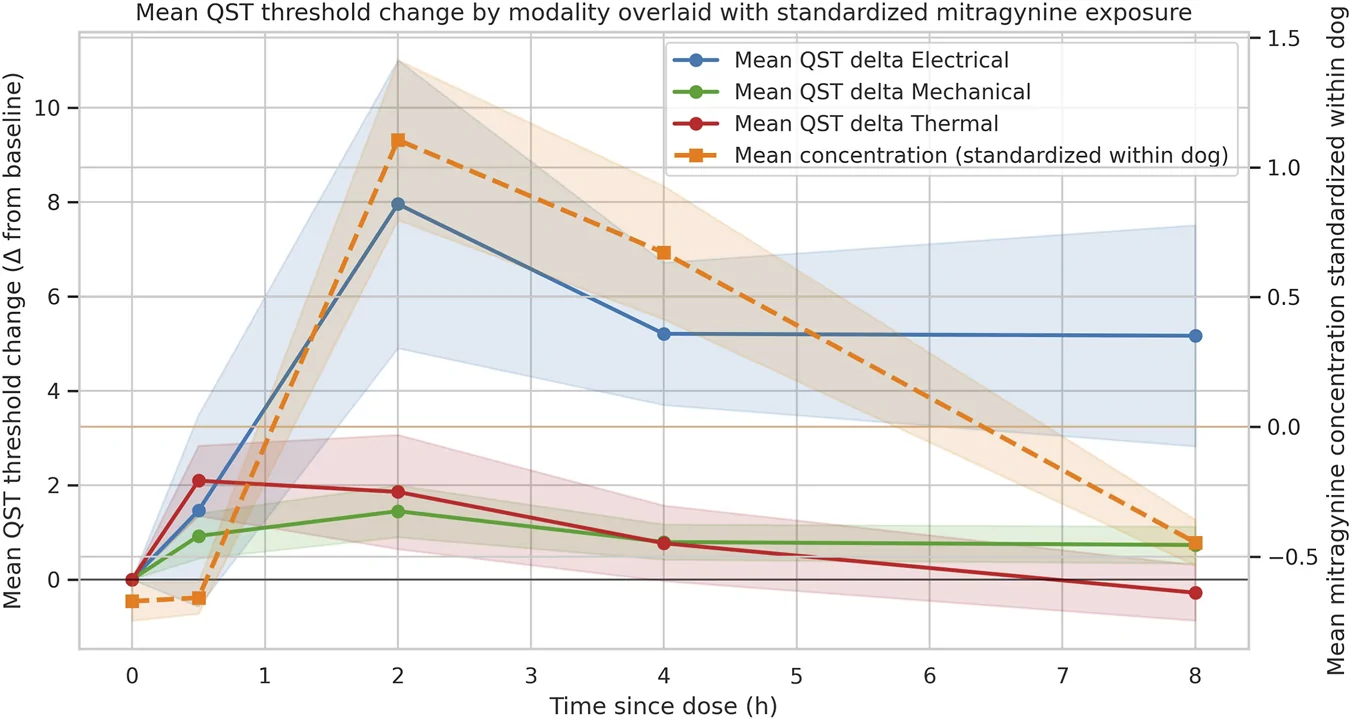
Mean QST threshold change (Δ from baseline) by modality overlaid with standardized mitragynine exposure. Mean QST threshold change is shown over time for electrical, mechanical, and thermal modalities at each post-dose timepoint following encapsulated kratom administration. Solid lines represent the across-dog mean Δ for each QST modality, with shaded bands indicating SEM. The dashed orange line represents mean mitragynine exposure after within-dog standardization (z-scores). For each dog, the mean mitragynine concentration across post-dose sampling time points was subtracted from each observed concentration, and the result was divided by that dog’s post-dose concentration standard deviation. The horizontal line at Δ = 0 marks baseline.

**FIGURE 6:**
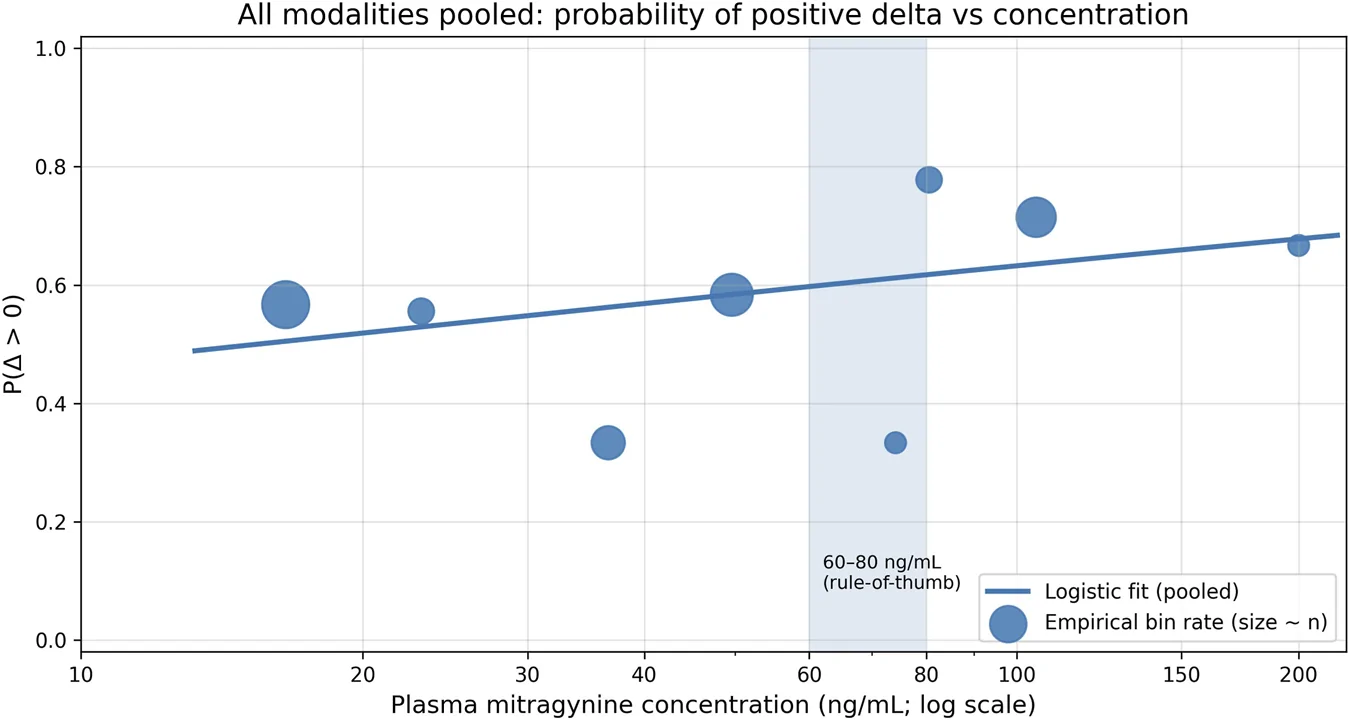
Relationship between plasma mitragynine concentration and probability of increased sensory threshold. Pooled quantitative sensory testing data across mechanical, thermal, and electrical modalities are shown as the probability of a positive change from baseline sensory threshold (Δ > 0) relative to plasma mitragynine concentration. Points represent empirical binned response rates, with point size proportional to the number of observations within each concentration bin. The solid line represents the pooled logistic fit. The shaded region indicates the approximate 60–80 ng/mL concentration range identified as a potential lower exposure range where the probability of a positive sensory threshold response begins to increase. The most consistently positive empirical response rates were observed at approximately 80–150 ng/mL, although substantial variability was present across modalities and individuals.

For visualization of the temporal relationship between mitragynine exposure and QST responses, plasma mitragynine concentrations were standardized within dog using z-scores; each concentration was transformed by subtracting that dog’s mean post-dose concentration and dividing by that dog’s post-dose concentration standard deviation. These standardized values were then averaged across dogs at each time point and were used only for graphical display (Figure 5). An exploratory pooled exposure-response analysis across all QST modalities was performed to evaluate whether plasma mitragynine concentrations were associated with an increased probability of a positive change in sensory thresholds from baseline (Figure 6). Because mechanical, thermal, and electrical QST assess distinct nociceptive and sensory processing pathways, this pooled analysis was interpreted as a global clinical response summary rather than a modality-specific PK-PD model. Although the relationship was variable and lacked a discrete threshold effect, the fitted logistic model demonstrated an overall positive concentration-response trend. Plasma mitragynine concentrations of approximately 60–80 ng/mL marked a reasonable lower range at which the probability of positive sensory threshold changes began to rise; however, observations in this interval were limited. The most consistent positive empirical responses occurred at concentrations of 80–150 ng/mL, where binned probabilities of positive delta rates were 0.71–0.78 (∼70–80%). These findings indicate that kratom exposure correlates with a greater likelihood of sensory threshold elevation, despite substantial inter-individual and modality-specific variability.

### 3.6 Safety

All complete blood count and serum biochemical parameters remained within clinically acceptable limits and were unremarkable across all treatment groups throughout the study period (Supplementary Tables S3 and S4). No adverse effects were observed in the placebo group. In the buprenorphine group, all eight dogs exhibited at least one adverse event within the 8-h observation period, including hypersalivation (8/8), vocalization (6/8), sedation/sleepiness (5/8), and trembling (4/8). In the kratom group, adverse effects were limited, with vocalization observed in two dogs and transient soft stool reported in 1 dog between 2 and 4 h post-administration. Additionally, two dogs demonstrated increased interaction, reduced fearfulness, and decreased frenetic behaviors; one dog was described as tranquil/calm, and two dogs exhibited no observable side effects.

## 4 Discussion

Across QST modalities, buprenorphine elicited the largest threshold increases, with group-average peaks exceeding modality-specific $MDC_{95}$ thresholds and high individual-dog responder rates (≥87.5%) in electrical, mechanical, and thermal tests; kratom produced modality-dependent effects, strongest in electrical QST (87.5% responders) but modest in thermal and mechanical. Using the current study design, buprenorphine exhibited broad-spectrum antinociceptive effects, while kratom produced modality-specific responses, predominantly affecting mechanical and electrical nociceptive thresholds, with minimal confounding sedation effects.

This differential efficacy across modalities suggests distinct mechanisms of action for each compound, warranting further investigation into their molecular targets and downstream signaling pathways. Modality-specific effects indicate that kratom may modulate descending inhibitory pathways or influence spinal cord processing more prominently than directly altering peripheral nociceptive input, a characteristic that distinguishes its analgesic action from that of buprenorphine, which demonstrates a more comprehensive influence on both peripheral and central pain mechanisms (Hanapi et al., 2021). Buprenorphine, a partial µ-opioid receptor agonist, has demonstrated antinociceptive effects across various pain models, including thermal and mechanical nociception, in both rodents and felines, further supporting its broad-spectrum action (Doodnaught et al., 2017; Steagall et al., 2006). In dogs, buprenorphine has also been shown to increase thermal and mechanical nociceptive thresholds, albeit with varying potency and duration depending on the route of administration (Stöckel, 2012). Conversely, the more selective antinociceptive profile of kratom, particularly its stronger effect on electrical thresholds, suggests a nuanced interaction with pain pathways that may not involve the broad opioid agonism characteristic of buprenorphine, thereby warranting further investigation into its specific receptor targets and downstream signaling cascades to fully elucidate its analgesic mechanism. Electrical thresholds were obtained using short pulse widths (20 µs) and high-frequency constant-current stimulation, which preferentially recruits large myelinated afferents (Kutafina et al., 2023). The larger electrical QST deltas relative to modest changes in gradual-ramp heat thresholds suggest kratom’s predominant measurable effect in this dataset is on fast-conducting afferent pathways and/or central sensory gating, rather than strong suppression of C-fiber heat nociception (Rukwied et al., 2020). Mechanical and thermal QST primarily interrogate peripheral nociceptor transduction, whereas electrical stimulation reflects central sensory processing; the preferential elevation of electrical thresholds observed with kratom suggests a centrally weighted antinociceptive profile, in contrast to the broad peripheral and central effects demonstrated by buprenorphine. Although the preferential increase in electrical thresholds observed in this study is suggestive of centrally mediated modulation of sensory processing, this interpretation should be considered hypothesis generating, and electrical QST is not definitive for identifying specific descending inhibitory mechanisms; therefore, future studies should incorporate targeted assessments such as conditioned pain modulation or diffuse noxious inhibitory control-like paradigms to more directly evaluate effects on endogenous pain inhibition, as this was not performed in the current study.

Preclinical findings suggesting that mitragynine, a primary alkaloid in kratom, modulates noradrenergic and serotonergic pathways and stimulates post-synaptic alpha-2 adrenergic receptors, which are known to influence fast-conducting afferent pathways (Chien, 2017). Such modulation could contribute to the observed changes in electrical thresholds, particularly given the role of alpha-2 adrenergic receptor activation in descending pain-inhibitory pathways and direct peripheral nerve excitability (Matsushita et al., 2021). Notably, alpha-2 adrenergic agonists are widely regarded as a gold standard in veterinary medicine for achieving potent, centrally mediated analgesia (Lemke, 2004; White et al., 2018). The observed pronounced elevation of electrical thresholds, may explain a plausible biologic framework where alpha-2 agonism modulates descending inhibitory pathways to reduce sensitivity to noxious stimuli. However the present study was not designed to directly test alpha-2 adrenergic involvement and future chronic pain models with alpha-2 antagonists should be investigated (Wei and Pertovaara, 1997; Kruegel et al., 2016; Reeve et al., 2020).

The complex pharmacology of kratom, involving over 25 alkaloids with diverse receptor affinities, including µ-, κ-, and δ-opioid receptors, as well as dopamine D1, serotonin, and adenosine-2a receptors, further supports the hypothesis that its analgesic profile extends beyond a simple opioid-like mechanism (Vento et al., 2021). Although kratom contains multiple bioactive alkaloids, the present study was not designed or powered to determine the contribution of individual non-mitragynine constituents to sensory threshold changes. Because non-mitragynine alkaloids and metabolites were low, mechanistic attribution to 7-hydroxymitragynine or other minor constituents would be speculative. Future studies using purified alkaloids or targeted pharmacodynamic modeling will be required to define the relative contribution of individual kratom constituents.

The pharmacokinetic profiles of kratom and buprenorphine appear closely linked to their distinct pharmacodynamic outcomes, suggesting that temporal variations in plasma concentrations play a key role in the onset and duration of antinociceptive effects. For kratom, mitragynine exhibits a rapid time to peak plasma concentration of approximately 2.4 h in healthy beagle dogs. In this study, the compound reached a maximum plasma concentration of 133.3 ng/mL following the third dose, with an elimination half-life (t_1/2_) of 18.7 h, and may contribute to the sustained elevation in electrical thresholds observed in this study, suggesting a pharmacokinetic-pharmacodynamic correlation for its centrally mediated antinociceptive effects. Conversely, the pharmacokinetic profile of buprenorphine, characterized by more rapid clearance, correlates with its broader, albeit shorter-lived, analgesic efficacy. These disparate profiles underscore the importance of integrating pharmacokinetic data with observed antinociceptive responses to better characterize the therapeutic potential of these agents.

Kratom was associated with fewer and less severe adverse effects compared to buprenorphine under the conditions of this study. While buprenorphine produced consistent and expected opioid-related side effects, kratom demonstrated a more limited adverse event profile, with some dogs exhibiting behavioral changes suggestive of improved comfort or reduced anxiety. Although these observations should be interpreted cautiously given the small sample size, they support the potential for kratom to provide analgesic benefit with a more favorable tolerability profile in comparison to traditional opioids.

Several limitations of this study must be acknowledged, including the reliance on behavioral endpoints for QST in dogs, which introduces subjectivity in interpreting conscious perception *versus* reflexive responses and may be influenced by individual temperament, anxiety, or feasibility challenges (Caddiell et al., 2023). Buprenorphine’s strong sedative effects may have affected the blinding process, as the profound sedation observed may have inadvertently cued observers to the treatment identity. This limitation was mitigated through standardized response definitions, investigator training, and consistent application of QST by a single experienced operator, improving reliability and reducing inter-observer variability. Although the crossover design increased efficiency by allowing each dog to serve as its own control, the small sample size limited power to detect modest treatment effects. So, while the study was adequately powered for detecting large treatment effects, such as buprenorphine *versus* placebo, it was underpowered for detecting smaller kratom-associated effects, particularly in thermal modalities. Based on observed paired treatment effects, detection of kratom effects across all QST modalities would require approximately 30 dogs, driven by the smaller and more variable thermal response. Additionally, evaluation of acute effects in healthy beagles rather than client-owned dogs with naturally occurring clinical pain limits translatability to diverse populations (Klinck et al., 2017). This approach was intentionally selected to minimize confounding variables and establish a controlled baseline for modality-specific pharmacodynamic effects, providing a necessary foundation for subsequent studies in dogs with naturally occurring pain. All dogs enrolled in this study were spayed females, which limits assessment of sex-related variability in sensory thresholds, analgesic response, and kratom alkaloid disposition. Sex-related differences in nociceptive processing, opioid pharmacodynamics, and drug metabolism have been documented across species, and therefore the findings of this exploratory study may not be fully generalizable to male dogs, intact dogs, or broader clinical populations. Finally, administration routes between oral encapsulated kratom and intramuscular buprenorphine were different; however, this approach reflects clinically relevant administration practices, and by including a positive control (buprenorphine), this facilitated meaningful comparative interpretation of analgesic effects.

## 5 Conclusion

This study provides a critical initial assessment of kratom’s analgesic potential through a comprehensive QST approach, revealing distinct pharmacodynamic profiles compared to buprenorphine. These findings hold substantial clinical and translational relevance, as QST in dogs demonstrates similarities in somatosensory changes and has translational utility for informing analgesic responses in humans due to shared somatosensory pathways and pathophysiological similarities in pain processing (Lascelles et al., 2022). Future investigations should aim to elucidate the specific mechanisms underlying kratom’s differential effects on electrical *versus* thermal and mechanical sensory thresholds, potentially through targeted receptor antagonism studies, detailed pharmacokinetic/pharmacodynamic modeling, and extension to client-owned dogs with naturally occurring pain conditions.

## Data Availability

The data presented in the study are deposited in the University of Florida Digital Collections repository and can be accessed at: https://ufdc.ufl.edu/ir00012409/00001.
