# Supplementary material for: Exploratory evaluation of encapsulated kratom using multimodal quantitative sensory testing in dogs
Source: Front Pharmacol. 2026 Sep 11;17:1895945. doi: 10.3389/fphar.2026.1895945 (PMC13612990; doi:10.3389/fphar.2026.1895945)
Supplement: Supplementary file 1 [file Table1.docx]

**Ultra-performance liquid chromatography-triple quadrupole mass spectrometer (UPLC-MS/MS) for the quantification of buprenorphine, mitragynine and its metabolites**

Two separate UPLC-MS/MS methods were employed for the quantification of buprenorphine, mitragynine, and their metabolites in canine plasma. Samples were analyzed using freshly prepared calibration and quality control standards following the U.S. Food and Drug Administration bioanalytical method validation guidelines (1). For buprenorphine and its metabolites, samples (25 µL) were processed with acetonitrile (100 µL) containing internal standard (IS, buprenorphine-*d*4, 5 µg/mL) and formic acid (0.05 %, v/v). For mitragynine, samples were processed with methanol containing IS (mitragynine-*d*3, 20 µg/mL). Samples were vortex-mixed, filtered through a 0.45 µm 96‑well Solvinert filter plate (Millipore, St. Louis, MO), centrifuged at 2000 rpm for 5 min at 4 °C, and injected into the Waters Acquity I coupled with Xevo® TQ-S micro mass spectrometer (UPLC-MS/MS) for both methods. The chromatographic separation for all the analytes was achieved using a linear gradient elution program using a Waters Acquity UPLC BEH C_18_ column (1.7 µm, 2.1 x 100 mm) with a VanGuard pre-column of a similar chemistry (Waters Co, Milford, MA), with flow rate of 0.35 mL/min. The aqueous phase comprised 0.1% formic acid in water (A) and the organic phase consisted of 0.1% formic acid in acetonitrile (B) and was utilized for the quantification of buprenorphine. The gradient program was initiated at 2% mobile phase B from 0 to 0.8 min, increase to 85% B between 0.8 and 2.0 min. It was held at 85% B until 3.0 min, after which it was back to 2% B from 3.1 to 3.5 min to allow column equilibration. For mitragynine, the aqueous phase comprised of 2.5 mM ammonium acetate buffer, pH 3.5 (A) and the organic phase consisted of acetonitrile (B). The mobile phase gradient was initiated at 20% B and maintained until 1.0 min, followed by a gradual increase to 23% B over the next 5 min. The gradient was then ramped up to 90% B between 6.0 and 8.0 min and maintained until 8.5 min. Re-equilibration was achieved by returning the system to 20% B from 8.5 to 9.0 min. The column oven was set to 50 °C, while the autosampler was maintained at 4 °C. A strong needle wash solution composed of water, acetonitrile, methanol, and isopropyl alcohol (1:1:1:1, v/v) containing 0.1% formic acid, whereas the weak needle wash consisted of water, acetonitrile, and methanol (2:1:1, v/v) with 0.1% formic acid. Data acquisition was accomplished using MassLynx software (version 4.2, Waters Corporation) and quantification was carried out using TargetLynx software. The optimized compound-specific parameters for UPLC–MS/MS analysis are summarized in Table S1.

**References**

1.	Analytical Procedures and Methods Validation for Drugs and Biologics Guidance for Industry. https://wwwfdagov/files/drugs/published/Analytical-Procedures-and-Methods-Validation-for-drugs-and-Biologics pdf.

**Table S1.** Compound parameters for quantification of analytes using UPLC-MS/MS methods

| Analytes | Parent (*m/z*) | Daughter (*m/z*) | Cone (V) | Collision V) |
| --- | --- | --- | --- | --- |
| Buprenorphine | 468.3 | 55.0 | 34 | 54 |
| Norbuprenorphine | 414.1 | 83.0 | 72 | 46 |
| Norbuprenorphine-3-glucuronide | 590.3 | 414.3 | 30 | 40 |
| Buprenorphine-3-glucuronide | 644.3 | 468.4 | 2 | 38 |
| Buprenorphine-*d*4 (IS) | 472.2 | 59.0 | 6 | 58 |
| Mitragynine | 399.2 | 174.2 | 60 | 32 |
| 7-Hydroxymitragynine | 415.19 | 190.1 | 42 | 30 |
| 9-hydroxycorynantheidine | 385.21 | 160.07 | 60 | 32 |
| Mitragynine acid | 385.21 | 174.16 | 60 | 32 |
| Mitragynine-*d*3 (IS) | 402.21 | 177.06 | 26 | 30 |
